# P06 Working differently – ‘Accepting Me’

**DOI:** 10.1093/rap/rkac067.006

**Published:** 2022-09-28

**Authors:** Lindsey Clarke

**Affiliations:** Alder Hey Children's Hospital, Liverpool, United Kingdom

## Abstract

**Introduction/Background:**

Management of children with chronic pain predominantly focuses on paced and graded activity to regain functional loss, this case summarises a different approach, resulting in improved patient outcome through use of ‘acceptance’.

Early intervention from therapeutic services is integral to patient’s prognosis with attention being given to restoring limb function, improving pain and increased QoL. The majority of people with CRPS will see a reduction in pain within two years. However, evidence supports some people will continue with pain despite treatment and therapy intervention. At which point is a different approach needed, enabling the individual to take control.

**Description/Method:**

Teenage female originally diagnosed with CRPS with developing chronic pain patten. Admitted for two weeks rehab session following goal setting using the COSA under the occupational therapy service. Initial goals met during inpatient stay; child discharged with advice to continue progress at home. Unfortunately, she regressed and subsequently functional level reduced. During follow up appointments it became clear that she was struggling with her perception of “being different” and not being able to complete the tasks she wanted to do.

Using elements from Ten footsteps approach the therapist was able to explore and empower, particularly, using “footstep 2” Acceptance. This enabled to the patient to acknowledge that pain was a present in her life however did not need to stop her living an active life with some adjustments. By spending some time exploring how wheelchair users can achieve and succeed, the patient was able to progress by accepting her limitations due to pain whilst finding ways to do the things that she wants and needs too. For example, she spent time education her school about how the environment and attitudes could be changed to help her be an active member of the school community. Furthermore, she accepted in the short term that using her wheelchair outdoors meant she could spend time with friends and remain independent at home with ADLS, empowering her to be in control of her pain levels.

Although other elements of the Ten Footsteps approach where used Acceptance was crucial to compliance with intervention and enable her to feel in control of her therapy input.

Therapy remains ongoing, however time between follow ups has increased significantly, with increased patient control of symptoms and their management. Furthermore, she actively participates in school activities and has become an advocate for herself and others in similar situations.

**Discussion/Results:**

This particular case, highlights the advantage to having Occupational Therapy treating in this area, being able to offer a holistic approach to intervention but also focus meaningful and purposeful occupation. This results in a balance of health and well-being being achieved, regardless of the individual functional deficits.

Occupational therapists are best placed to lead with this patient group due to their uniquely training to be able to bridge the gap between physical health and mental health.

This highlights how both are intertwined together and neither can be separated when aiming to achieve a meaningful and purposeful life.

Furthermore, there is the potential for occupational therapy to bridge the gap between MDT member ensuring continuity for the patient.

The Initial treatment programme failed to yield the results and the patient plateaued it was important to explore if this was the right approach always rehab or is there a place for Acceptance - resulting in a fulfilled meaningful and purpose full life.

This case also highlights how using the evidence base within Occupational Therapy alternative approach can be used depending on the individual that is being treated. In this case acceptance of a “life with pain” and a life using a wheelchair enable the patient to accept their functional abilities and work with them to achieve a meaningful and purposeful life. Furthermore, this enable the patient to progress physically which with the traditional approach of returning to baseline (pre illness) had been halted.

Until OT discussed living life to the full regardless of adaptations and equipment the patient was reluctant to work on graded goals as they wanted to achieve their ultimate goal of walking without an aid. However once focusing on success stories of individuals who live a fulfilled life despite of a disability the patient was able to refocus and achieve.

**Key learning points/Conclusion:**

Sometimes the well-used approach is the most comfortable for us as therapists however this often results in a one size fits all intervention and will not achieve the best outcome therefore alternatives should always be explored.

A holistic approach is needed in order to enable patients to reach their goals. There is not a one size fits all approach and as an Occupational therapist we have a duty to explore and adapt in order to get the best outcomes for our patients.

Acceptance was the key for the patient to be able to explore current abilities, identity deficits and work on individual goals within their capabilities. Therefore, what was evident is that by accepting current abilities the patient was able to work on more realistic and grade goals – resulting in progress`.

For future practice exploring a holistic and realistic approach need to be embraced from the beginning with a view to be flexible. Standard needs to be widened to ensure that therapist feel comfortable when the “norms” are not working and know how to adapt and negotiate difficult conversations.

